# Effectiveness of Training in Evidence-Based Practice on the Development of Communicative Skills in Nursing Students: A Quasi-Experimental Design

**DOI:** 10.3390/healthcare12181895

**Published:** 2024-09-21

**Authors:** María Ruzafa-Martínez, Verónica Pérez-Muñoz, María Belén Conesa-Ferrer, Antonio Jesús Ramos-Morcillo, Alonso Molina-Rodríguez

**Affiliations:** 1 Department of Nursing, Faculty of Nursing, University of Murcia, Av. Buenavista, 32, El Palmar, 30120 Murcia, Spain; maruzafa@um.es (M.R.-M.); mb.conesaferrer@um.es (M.B.C.-F.); ajramos@um.es (A.J.R.-M.); alonso.molina@um.es (A.M.-R.); 2 Department of Sociosanitary Care, Faculty of Social and Health Sciences, University of Murcia, Av. De las Fuerzas Armadas, s/n, 30800 Lorca, Spain

**Keywords:** communication, attitudes, evidence-based practice, nursing education

## Abstract

Background: Communication skills (CSs) and evidence-based practice (EBP) are key foundations for offering personalized and quality nursing care. CS that results in open communication is fundamental for applying EBP. Objectives: The objective of the study was to assess the relationship between the acquisition of EBP competencies and learning CS after taking an EBP course in the nursing degree. Methods: Pre-test-post-test quasi-experimental study without a control group. The study population comprised fourth-year undergraduate nursing students. Before and after an EBP course, students completed a questionnaire on CS competence (HP-CSS) and EBP competence (EBP-COQ). The EBP course lasts for 15 weeks, with 150 h of work for the student. Out of these 150 h, 60 are conducted in person, while the remaining 90 h are for independent or group work, utilizing the flipped classroom methodology. Bivariate, correlational, pairwise Student’s *t*-test, and linear regression multivariate analyses were performed. Results: The sample was composed of 153 students. After completing the EBP course, there was a statistically significant improvement in informative communication (*p* = 0.046) and assertiveness (*p* = 0.018). However, there were no observed changes in empathy, respect, and the total score of the CS competence. The results from the multivariate analysis showed that the dimensions of attitude towards EBP and EBP knowledge had a positive relation to three of the four dimensions of the CS and overall competence. Regarding the students’ variables and characteristics, admission through special programs for athletes and older students (*p* < 0.001) and being female (*p* = 0.004) were positively statistically associated with empathy. Conclusions: EBP training positively affects the development of CS in nursing students, as shown by significant improvements in the total scores of the CS and the dimensions of informative communication and assertiveness after the intervention. This study demonstrates the initial applicability and usefulness of an EBP training program for the development of CS in nursing students.

## 1. Introduction

Communication is central to each interpersonal interaction around health care [1]. Many authors in nursing, such as Hildegar Peplau and Joyce Travelbee, have underlined the importance of communication in interpersonal relations [2]. Peplau, in her theory of interpersonal relations, states that the nurse–patient relationship is characterized by linguistic-therapeutic communication [3]. Travelbee, on her part, integrated communication and empathy as elements necessary in relationships between human beings. Also, the problem resolution between nurses and patients approach proposed by Abdellah highlights the importance of promoting verbal and non-verbal communication between nurses and patients to guarantee high-quality care [4]. Updated communication conceptual frameworks expand the value of defining communication beyond information exchange by including information creation through the development of shared understanding [5].

The American Association of Colleges of Nursing affirms that effective communication skills (CSs) are fundamental for nursing students to provide safe patient care [6]. Facilitating their application in the work environment and the personal sphere is one of the key cross-cutting competencies in social health sciences [7]. Also, there is a general consensus that the incorporation of CS leads to personalized attention and must, therefore, be part of the professional practice of the nurses [8], which further underlines their importance in the area of healthcare.

The existing evidence shows that nursing professionals who possess adequate CS offer quality care centered on patients [9,10,11,12,13,14,15]. It has been shown that establishing therapeutic communication between nurses and patients is essential for providing quality care [16]. This translates into positive results in the mood and satisfaction of patients, in adherence to the treatment, and the improvement of quality indicators in healthcare [17,18,19,20]. On the contrary, inefficient communication can lead to preventable mistakes, delays in treatment, or even adverse effects on patients [21]. In addition, health professionals with CS training gain benefits as their confidence in the management of communication in complex contexts increases, such as giving bad news, fielding difficult questions, and responding to situations with a high emotional load [22].

CSs in health professionals are described as “the set of verbal and non-verbal techniques and behaviors that shape the relational competencies of health professionals through which they express, in an interpersonal context (patient-centered) and a specific situation, their needs, feelings, preferences, opinions, desires, and rights, resulting in a series of consequences in the relationship that affect the patient themselves, the professionals, their relationship, and even the health system” [23]. CS encompasses diverse essential dimensions, such as informative communication, active listening, empathy, respect, authenticity or congruency, and assertiveness [22,24]. These elements play a crucial role, simultaneously acting as facilitators or barriers that can either promote or make difficult, efficient communication with the patient [25].

The background on the subject indicates that empathy is a dimension that can be acquired, and it is expected that nursing professionals incorporate it as a fundamental pillar in the nurse–patient relationship [26,27]. This dimension has been positively related to emotional intelligence, CS, and the ability to provide care to nursing students [28]. It has also been observed that those with higher levels of CS have greater resilience [29]. The relationships between the CS and the sociodemographic characteristics of students enrolled in health sciences have been explored. It was observed that the age of the undergraduate and graduate nursing students was positively correlated with their attitude toward learning communication skills [30]. With respect to sex, it seems that women have higher levels of affective and cognitive attitudes as compared to their male counterparts [31].

Communication is not an innate skill that varies from person to person. However, it can be acquired through training and experience [13]. Along this line, many studies coincide in that CS training in nursing professionals and students is inadequate [21,32], in part due to the lack of importance given to non-technical or soft skills, as compared to technical competencies [18,24]. Learning technical skills has traditionally been the basis on which the content of the health sciences training programs is developed, while the rest of the soft skills, such as CS, leadership, or teamwork, are not generally included in nursing degree teaching plans [33]. It has been shown that education interventions on communication skills that use diverse techniques, such as simulation-based training, hybrid methods, and cooperative learning, can strengthen the communication skills of nurses and nursing students [18,34]. The most promising result suggests that education interventions, particularly those that employ personalized feedback approaches, tend to improve students’ communication skills most effectively [35].

Presently, the relevance of acquiring CS and evidence-based practice (EBP) skills as key foundations for providing personalized and quality nursing care is emphasized [36]. The intention of EBP is to integrate the best external evidence with the clinical experience of professionals, considering the patient’s choice and the available resources [37]. The third component of EBP implies having in mind the preferences and values of patients when making clinical decisions [38], and therefore acquiring CS that facilitates open communication, in which the patient feels recognized, and to the highest degree possible, is able to actively participate in the healthcare process, is fundamental for the application of EBP [39].

Recent students have concluded that nurses who apply care practices based on evidence dedicate more time to discovering patients’ preferences and utilize CS to improve professional care [8]. Also, a positive attitude toward learning communication competencies leads to a positive attitude toward EBP [30]. Research indicates a strong relationship between communication skills, EBP, and effective healthcare delivery. Communication skills and EBP competencies predict nursing students’ performance in simulated clinical scenarios [40]. Integrating patient values into EBP through effective communication and shared decision-making is particularly important for chronic pain conditions, as it leads to better outcomes than traditional biomedical approaches [41]. There is a significant link between evidence-based practice (EBP) and communication skills (CSs) in the training of nursing students. However, no studies have investigated their relationship in the learning process. It is crucial to explore the connection between EBP and communication skills in nursing students to enhance nursing education and improve patient outcomes through more effective, evidence-based care. In the present study, the initial hypothesis is that EBP training is positively related to the learning of CS. For this, the aim of the study was to assess the relationship between the acquisition of EBP competencies and learning CS in nursing students after taking an EBP course in the nursing degree.

## 2. Materials and Methods

### 2.1. Design

A quasi-experimental pre-post study was conducted with undergraduate nursing students without a control group.

### 2.2. Environment and Participants

The study was conducted in the Faculty of Nursing at the University of Murcia during the 2022/23 academic year. The study population consisted of 180 students in their fourth year enrolled in the Clinical Practice and Evidence-based Nursing course of the nursing degree program.

During the academic year, all the students received the same training, including the EBP course, high-fidelity clinical simulation, and 325 h of clinical practice.

### 2.3. Sample Selection

The sample size calculation was based on the paired Student’s *t*-test [42]. For a quasi-experimental study with only one group (experimental group), the required sample size is approximately 128 subjects. The study assumes a standard deviation of 8.04 [43], a minimal detectable difference of 0.25 in the HP-SCC total competence score, 80% power, and a significance level of 0.05 (two-tailed).

The sampling method used was non-probabilistic, and no random sequence was obtained. The entire sample comprised nursing students who had taken the evidence-based practice (EBP) course, meaning that all groups of participants underwent the intervention. The study included students enrolled in the nursing degree program who had completed the EBP course. Students who had not completed the EBP course or who were part of an ERASMUS or other student mobility program were excluded from the study.

### 2.4. Intervention

The nursing degree program is a four-year academic training that includes a mandatory subject called Clinical Practice and Evidence-based Nursing in the final year. This subject is taught during the first term, which is from September to December, and lasts for 15 weeks, with 150 h of work for the student. Out of these 150 h, 60 are conducted in person, while the remaining 90 h are for independent or group work, utilizing the flipped classroom methodology. The students are divided into groups of 40 to attend seminars where they cover the course content and into groups of 20 for critical appraisal laboratories. The content of the course includes the four main steps of the EBP process: cultivate a spirit of inquiry, ask the clinical question in PICOT format, search for the best evidence, and critically appraise the evidence. The students review the content of these modules in online materials provided by the lectures for the preclass asynchronous activities before the face-to-face seminars and laboratories. A detailed description of the content is shown in Table 1.

### 2.5. Variables and Data Collection Instruments

The data collection document included the following sociodemographic variables: age, gender, method of admission to the degree, previous level of education, and class attendance. Variables on technology training and use included training before EBP, training on the EBP methodology, reading of articles, using Twitter, Facebook, and Instagram to consult scientific evidence, using health blogs to consult scientific evidence, and reading networks to consult scientific evidence.

EBP competence was assessed with the previously validated EBP Competence Questionnaire for nursing students (EBP-COQ) [44]. This tool has been validated and used in various countries beyond Spain, including Italy, Turkey, Greece, and Poland [45,46,47,48]. The EBP-COQ was specifically created to assess the self-perceived EBP competence of nursing students. The overall Cronbach’s alpha of the questionnaire was 0.888. Good reliability scores were also obtained in the three dimensions that shape the questionnaire: dimension 1 “Attitude towards EBP”, with Cronbach’s alpha of 0.940, dimension 2 “EBP skills”, with a Cronbach’s alpha of 0.756, and dimension 3 “EBP knowledge”, with a Cronbach’s alpha of 0.800. The EBP-COQ includes 25 questions. It is answered with responses ranging from 1 to 5 (1 is the lowest level, and 5 is the highest) for the 3 dimensions and the overall EBP competence. The maximum total score of the EBP-COQ provides an overall index of “EBP Competence”, allowing the identification of a high level of competence (maximum questionnaire score: 5).

Main outcome: To assess communication skills, the Health Professionals Communication Skills Scale (HP-CSS) was used and validated to assess the self-perceived CS of health professionals [49] and students [43]. Up to now, this tool has been adapted to Chinese [50]. The Cronbach’s alpha of the scale was 0.88. Good reliability results were also obtained for the 4 dimensions that shape the questionnaire. Dimension 1, “Informative communication”, assesses the way to obtain and provide information with respect to patients, and obtained a Cronbach’s alpha of 0.78. Dimension 2, “Empathy”, reflects the ability to understand patients’ feelings and makes it evident through active listening and emphatic answers, with a Cronbach’s alpha of 0.77. Dimension 3, “Respect”, assesses respect in the clinical relationship that is established between patients, with a Cronbach’s alpha of 0.74. Dimension 4, “Assertiveness”, reflects the ability to be assertive or have socially skillful behaviors in the clinical relationship with patients, with a Cronbach’s alpha of 0.65. The HP-CSS is composed of 18 items, with a response range of 1 to 6 (1 is the lowest level and 6 is the highest). Dimension 1 is scored with 6–36 points and includes 6 items, dimension 2 is scored with 5–30 points and includes 5 items, dimension 3 is scored with 3–18 points, and includes 3 items, and dimension 4 is scored with 4 to 24 points and includes 4 items. Meanwhile, the maximum total score of the HP-CSS is 108.

Both the HP-CSS and EBP-COQ tools have been previously used in the Spanish context, demonstrating a strong association between EBP competence and CS [40].

An online data collection notebook was designed, and the answers were obtained before the start of the course, in September 2022, and at the end of the first term, when the EBP course ended, in January 2023.

### 2.6. Data Analysis

The qualitative variables were expressed as frequencies and percentages. The mean and standard deviation (SD) were calculated for the quantitative variables. Pearson’s R coefficient was used to study the relationship between the continuous variables. For dichotomous and polychotomous variables, the Chi-square test and a one-way ANOVA were performed, respectively. To compare the change in communication skills and EBP through time, the parametric paired samples *t*-test was utilized. The difference in means (DM) was used to measure the effect with 95% confidence intervals (95%CI) on the scores of the dimensions studied. Cohen’s d was used to obtain the effect size, through the use of values proposed by Ferguson, where 0.41 indicated a small effect, 1.15 indicated a moderate effect, and 2.70 indicated a large effect [51]. A multivariate model (forward stepwise multiple linear regression analysis) was created to calculate the magnitude of the effect of the characteristics of the students and the EBP competence they showed before its association with communication skills. In the statistical analysis, a level of significance of 5% (*p* ≤ 0.05) was considered. The data were analyzed using the statistical package Jamovi v.2.3.19.0 for Mac.

### 2.7. Ethical Aspects

The study was approved by the Ethics Committee of the University of Murcia (256/18). The ethical approval of our study aligns with broader international guidelines, such as those established by the World Medical Association (WMA), specifically in the Declaration of Helsinki. This document sets forth fundamental ethical principles for medical research involving human subjects, emphasizing the need to respect the dignity, rights, and well-being of participants. By obtaining ethical approval, we ensure that our study adheres to these international standards, guaranteeing the protection of participants and the integrity of the research process.

The students’ participation was voluntary after an explanation of the study objective and the ethical guarantees was given. The anonymity of the students was maintained, and the confidentiality of the data obtained was ensured through the creation of a personal code.

## 3. Results

The final sample of the study comprised 153 fourth-year students from the nursing degree program (response rate of 85%). The students had a mean age of 24.2 years (SD = 8.05), and 78.4% were women. Access to the nursing degree was higher for students coming from high school (77.1%), and many students attended more than 50% of the classes. Students had no previous EBP and research methodology training, and 74.5% used social media to consult scientific evidence (Table 2).

The mean score in all the dimensions and total competence in EBP improved significantly (*p* < 0.001) in all nursing students after taking the EBP course. A statistically significant improvement was also observed in the dimension of informative communication (*p* = 0.046) and assertiveness (*p* = 0.018), with an increase of 0.53 and 0.60 points, respectively, after receiving the training (Table 3).

The analyses showed a positive correlation between the EBP-COQ and most HP-CSS dimensions (Table 4).

Being a woman and being admitted to the nursing degree through special admissions was also statistically significant with a greater score in the dimension of empathy (*p* = 0.010). Consulting social networks to obtain scientific evidence was associated with a higher score in all the dimensions and the total score of the CS (Table 5).

The results from the multivariate analysis showed that the dimensions of attitude towards EBP and EBP knowledge had a positive effect on three of the four dimensions of the CS and overall competency. The attitude towards EBP was also positively related to assertiveness. With respect to the variables and characteristics of the students, only being admitted to the degree through special admissions given to athletes and older students, and being a woman, were positively associated with empathy (Table 6).

## 4. Discussion

The aim of this study was to investigate if EBP training had a beneficial impact on the development of CS in nursing students. The study was performed with fourth-year students, whose sociodemographic characteristics were similar to other national (Spain) and international studies [8,31,52,53]. This facilitates the extrapolation of the findings obtained, thus broadening the external validity of the results. The main results show that after the intervention, positive and significant changes were observed in the total CS score and in the dimensions of informative communication and assertiveness. Also, the dimension’s attitude towards EBP and EBP knowledge showed a positive relationship with the CS dimensions and total score of the scale.

High mean scores were obtained in all the dimensions of the HP-CSS in the initial measurement of the study. The dimensions of the CS with the highest scores, according to the ranges for each dimension, were informative communication, empathy, and respect, as well as the total score of the scale. On the other hand, assertiveness obtained a moderate score. Similar results with the same instrument were obtained in an observational study performed with nursing students in Spain [43]. Scores that were somewhat lower among health professionals in Spain, including nurses, doctors, and nursing assistants, have also been observed [49]. On the other hand, a study conducted with health professionals in Turkey with the same instrument revealed slightly higher scores [54]. These disparities could be related to the accumulated work experience, the specific cultural norms in each country, and the variations in work environments.

Our findings indicate a significant improvement in assertiveness after the EBP training interventions, which is positively related to attitude towards EBP. Although no previous studies with similar interventions have been found, a recent systematic review of the literature revealed that approximately 64% of the specific education interventions on assertiveness, which utilized diverse teaching methods, such as simulation-based learning, classroom learning, peer support, and hybrid learning, were able to improve the capacity of assertive communication in nursing students and nurses [34]. Improving assertiveness is essential for preventing mistakes and improving the safety of the patient in healthcare environments [55]. It is essential for nurses to develop assertive communication skills to openly express their worries when providing care. The improvement in the attitude towards EBP rises as a facilitating factor in the learning process, perhaps when improving the perception of students on their ability to make decisions.

Likewise, an increase was observed in the informative communication dimension after the education intervention, which was associated with attitudes towards EBP and EBP knowledge. These dimensions describe the way in which health professionals obtain and share information with respect to the clinical relationship they establish with patients. This includes the ability to communicate with patients, colleagues, and other health personnel [23]. The research showed that nurses struggle to prioritize dialogue with patients due to personal fears and shortcomings [56]. One of the strengths of EBP is that it provides tools for making decisions founded on scientific knowledge, and can favor more intimate and therapeutic communication. Nurses have positively valued this aspect, as it provides greater confidence and trust in their interaction with patients [57].

An improvement was also observed in the total score of the communication skills scale after the educational intervention, being positively related to attitude towards EBP and EBP knowledge. The EBP training program used the flipped classroom as the education methodology, where the students must become involved in self-learning before attending in-person classes. Also, learning about EBP and the approach utilized, which entails asking clinical questions that the students must answer by searching for research results and their interpretation, promotes critical thinking and judgment skills, key aspects in the acquisition of communication skills, as observed in other educational interventions [58]. In a similar manner, other teaching methodologies, such as the objective evaluation of standardized patient assessment, which also requires clinical judgment, have also shown positive results in the acquisition of communication competencies in nursing students [59].

Although the dimensions of empathy and respect showed a slight decrease in their scores after the EBP course, it must be underlined that these scores were still high, and the decrease was not statistically significant. These results align with previous studies, in which a similar trend was observed in the decrease in empathy among nursing students as they advance in their education [60,61]. This trend could be attributed to the students having clinical practices during the term, which provides them with a direct experience with patients. As other studies have pointed out, empathy decreases when one has experience in the clinical context with illnesses and suffering [61]. Likewise, it has been observed that empathy creates fatigue as students partake in clinical practice sessions, resulting in a decrease [62]. In any case, empathy, defined as the ability to understand the patient, is a complex construct composed of two components, a cognitive one, and an affective one [63]. An experimental study showed that cognitive empathy improved in nurses after an intervention that consisted of applying evidence-based practices to improve the therapeutic relationship with the patients [64]. It is possible that EBP training in students, uniquely developed in the academic context, may lack the specific resources to effectively promote empathy and respect, which underlines the need for this training to be transferred to the clinical context.

As for the sociodemographic and educational variables of the students, only gender and the method for being admitted to the degree showed a significant relationship with CS. Gender differences in empathy suggest that women might be naturally more attuned to others’ emotions, a quality beneficial in nursing. In this sense, it has been observed that empathy significantly improves in women, a finding that is supported by previous studies [65,66,67] but further research is needed to explore the underlying mechanisms. Likewise, the students who were admitted to the degree due to special admissions had a higher level of empathy. This percentage of the sample was composed of students who were admitted to the degree after vocational training, tests for those older than 25 and 45, and those who had a previous university degree. The existing literature supports the idea that the levels of empathy could vary depending on individual characteristics, such as gender, age, and self-esteem [68]. The positive association with special admissions raises questions about the role of diverse life experiences in developing empathy. Future studies should examine how different admission pathways contribute to empathy development.

The results from our study indicate that training and development of EBP competencies in nursing students could become a solid base of knowledge and skills, with a positive impact on their CS more specifically, informative communication and assertiveness. Clear and assertive communication between health professionals and patients could lead to a greater understanding of treatments, higher rates of adherence, and more positive health results. EBP training and learning communication skills are two competencies that feed into the training of nursing students [69]. The findings from the study support the importance of including formal education on EBP in nursing education programs, due to its effect on communication competency. This should result in a stronger emphasis on integrating EBP in nursing study plans, and the creation of additional resources and supports to facilitate learning and teaching of EBP in clinical practice.

### Study Limitations

The main limitation of the study was the absence of a control group. This lack of a control group may affect the internal validity of the study, as it makes it difficult to attribute the results exclusively to the intervention. The students, aside from EBP training, participated in high-fidelity clinical simulations and took part in clinical practices, so the independent effect of the EBP-specific intervention is unknown. On the other hand, the data related to EBP competence and CS were collected through self-reported instruments, which may lead to social desirability bias, although to avoid this, instruments were used that showed adequate internal validity in the Spanish context. The study was conducted only in a single institution. Therefore, further studies need to be conducted in other institutions with a control group or conducting a longitudinal follow-up to confirm our findings. Additionally, exploring the effects of cultural diversity and gender, or other sociodemographic differences, on communication skills can reveal variations in communication styles among nursing students. This evidence could help tailor the program to the different students.

## 5. Conclusions

EBP training had a positive effect on the development of communication skills of nursing students, as evidenced by significant improvements in the total CS scores and the dimensions of informative communication and assertiveness after the intervention.

This study demonstrates the initial applicability and efficacy of an EBP training program for the development of CS in nursing students, especially related to the dimensions of attitude towards EBP and EBP knowledge, which suggests its usefulness as an educational component in this area.

These results further promote the importance of establishing the regulated teaching of EBP in nursing student education programs.

To integrate EBP and CS training into nursing curricula, it would be interesting to incorporate modules dedicated to EBP and communication from the early stages of the program, the use of laboratories and clinical simulations that allow students to apply this knowledge in controlled environments, and the implementation of continuing education programs for teachers, thus ensuring that they remain up to date in these areas. In addition, the application of formative assessments with continuous feedback is suggested to measure and support student progress. Finally, the inclusion of interprofessional projects that encourage collaboration between different health professionals could further strengthen training in these essential competencies.

## Figures and Tables

**Table 1:** Description of the evidence-based practice (EBP) course.

CLASS Hours per Student	Working Hours per Student	Methodology	Content	Assignment
10	10	Seminar	Introduction to EBP. The significance of EBP in the development of nursing science General Concepts: - Nursing variability - Steps of the EBP - Magnitude, validity, bias Hierarchy of evidence and Grades of recommendation	Study of the contents with documentary and reference resources
5	5	Laboratory	Clinical question formulation using the PICO format	Formulate a Clinical PICO question
5	10	Laboratory	Designing and conducting the search for evidence in multiple evidence databases Evidence search hierarchy: - meta search engine: Trip Database, Epistemonikos, Evidence Portal, Evidence Search - Online Databases of Clinical Practice Guidelines: NGC, NICE, SIGN, RNAO, Guiasalud - Systematic reviews databases: Cochrane Library, JBI, CRD Search strategies: - controlled vocabulary (thesaurus/MeSH), keywords, boolean operators, limit function Searching databases: PubMed/Medline, CINAHL, PsycINFO^®^	Identify clinical practice guidelines, a systematic review, and an original study on a PICO question Describe the search hierarchy for the clinical questions. Detail the search strategies (controlled vocabulary, keywords, limit function and Boolean operators)
30	50	Seminar	Studies design: - Cross-sectional, case–control, cohort, controlled trial, systematic review, Clinical Practice Guidelines How to evaluate the evidence (critical appraisal using CASP tools, AGREE II) Applying the evidence to a patient care decision	Each week the students must read and critically appraise a paper (in total 6 documents) using CASP tools and AGREE II. Discussion of the paper in groups (up to 5 students) Presentation and discussion in a seminar with the teacher.
10	15	Laboratory	Final project: Response to a PICO question formulated based on a clinical scenario	In groups of up to 5 students, they must: identify a nursing problem in patients cared for during clinical training; formulate a PICO question; identify systematic reviews and/or primary articles; critically appraise search results; describe recommendations on the clinical question, and identify the level of evidence and grade of recommendation. Present the results of the final exercise in a poster to the laboratory group, giving reasons for implementation of the search results.

**Table 2:** Description of sociodemographic variables, training, and use of networks.

Age in years (Mean; SD)		24.2 (8.05)
Gender % (N)	Female	78.4% (120)
	Male	21.6% (33)
Method of admission % (N)	High School	77.1% (118)
	Vocational training	14.4% (22)
	Special admission	8.5% (13)
Other education % (N)	None	75.2% (115)
	Vocational training	19% (29)
	5-year bachelors	0.7% (1)
	4-year bachelors	3.3% (5)
	Master’s	2% (3)
Class attendance % (N)	<24%	3.3% (5)
	24–49%	9.8% (15)
	50–74%	30.7% (47)
	>75%	56.2% (86)
EBP training % (N)	None	88.2% (135)
	<40 h	5.2% (8)
	40–150 h	4.6% (7)
	>150 h	2% (3)
Training on Scientific Methodology % (N)	None	91.5% (140)
	<40 h	3.9% (6)
	40–150 h	3.9% (6)
	>150 h	0.7% (1)
Reading of articles per month % (N)	None	2.0% (3)
	1–3 articles	34.0% (52)
	>3 articles	64.1% (98)
Twitter/Facebook/IG % (N)	Yes	74.5% (114)
	No	25.5% (39)
Reading of social networks % (N)	Never	9.8% (15)
	Occasionally	32.0% (49)
	Monthly	13.1% (20)
	Weekly	33.3% (51)
	Daily	11.8% (18)

SD = Standard Deviation.

**Table 3:** EBP-COQ and HP-CSS scores before and after the EBP course.

	Mean (SD)		Difference in Means	95%CI		Cohen’s d	*p*
	Pre	Post		Lower Limit	Upper Limit		
Attitude EBP	3.78 (0.24)	4.36 (0.43)	−0.585	−0.655	−0.51570	−1.3445	<0.001
EBP Skills	3.10 (0.28)	4.00 (0.56)	−0.904	−1.008	−0.80051	−1.3936	<0.001
EBP Knowledge	2.88 (0.38)	4.20 (0.48)	−1.319	−1.416	−1.22256	−2.1810	<0.001
Total EBP	3.25 (0.17)	4.19 (0.42)	−0.936	−1.007	−0.86544	−2.1140	<0.001
CS Informative Comm.	30.78 (3.11)	31.32 (3.27)	−0.536	−1.063	−0.00905	−0.1625	0.046
CS Empathy	26.66 (2.90)	26.50 (3.17)	0.163	−0.266	0.59326	0.0607	0.454
CS Respect	16.93 (1.41)	16.77 (1.65)	0.163	−0.113	0.43972	0.0945	0.245
CS Assertiveness	15.84 (3.25)	16.44 (3.38)	−0.601	−1.100	−0.10245	−0.1925	0.018
Total CS	90.22 (8.14)	91.03 (8.78)	−0.810	−2.054	0.43283	−0.1041	0.200

CS = Communication Skills; CI = Confidence Interval; *p* = P valor.

**Table 4:** Correlation of sociodemographic variables and EBP-COQ with HP-CSS.

	Informative Communication		Empathy		Respect		Assertiveness		Total CS	
	R	*p*	R	*p*	R	*p*	R	*p*	R	*p*
Age	0.046	0.572	0.142	0.081	0.017	0.839	0.050	0.543	0.091	0.266
Attitude EBP	0.491	<0.001 ***	0.381	<0.001 ***	0.378	<0.001 ***	0.168	0.038 *	0.456	<0.001 ***
EBP Skills	0.461	<0.001 ***	0.332	<0.001 ***	0.395	<0.001 ***	0.081	0.321	0.397	<0.001 ***
EBP Knowledge	0.469	<0.001 ***	0.343	<0.001 ***	0.406	<0.001 ***	0.165	0.041 *	0.439	<0.001 ***
Total EBP	0.553	<0.001 ***	0.410	<0.001 ***	0.460	<0.001 ***	0.157	0.053	0.501	<0.001 ***

R = Pearson’s coefficient; *p* = P valor. * *p* < 0.05; *** *p* < 0.001.

**Table 5:** Relationship between sociodemographic variables, training and use of networks and HP-CSS.

		Informative Comm.		Empathy		Respect		Assertiveness		Total CS	
		Mean (SD)	*p*	Mean (SD)	*p*	Mean (SD)	*p*	Mean (SD)	*p*	Mean (SD)	*p*
Gender	Female	31.5 (3.03)	0.111	26.8 (3.07)	0.010 *	16.9 (1.57)	0.086	16.2 (3.29)	0.100	91.5 (8.34)	0.228
	Male	30.5 (4.00)		25.2 (3.26)		16.3 (1.88)		17.3 (3.64)		89.4 (10.21)	
Method of admission	High School	31.2 (3.41)	0.508	26.3 (3.15)	0.021 *	16.8 (1.67)	0.516	16.2 (3.43)	0.297	90.6 (9.08)	0.131
	Vocational training	31.2 (2.75)		26.2 (3.50)		16.5 (1.77)		17.2 (3.16)		91.2 (7.81)	
	Special admission	32.2 (2.86)		28.4 (2.26)		17.2 (1.34)		17.2 (3.32)		95.0 (6.92)	
Other education	None	31.3 (3.39)	0.997	26.5 (3.11)	0.866	16.8 (1.69)	0.760	16.3 (3.54)	0.809	90.8 (9.17)	0.921
	Vocational training	31.3 (2.94)		26.9 (3.40)		16.8 (1.63)		17.0 (3.13)		92.0 (7.57)	
	5-year bachelors	31.5 (3.78)		25.8 (3.81)		16.1 (1.47)		16.5 (2.51)		90.0 (9.75)	
	4-year bachelors	31.7 (1.15)		26.0 (3.61)		17.3 (0.57)		16.3 (0.57)		91.3 (4.51)	
Previous EBP training	None	31.3 (3.34)	0.165	26.4 (3.21)	0.449	16.7 (1.70)	0.337	16.5 (3.48)	0.875	91.0 (9.10)	0.740
	<40 h	30.3 (2.71)		27.9 (2.41)		16.9 (1.24)		16.3 (3.01)		91.3 (5.57)	
	40–150 h	33.4 (2.37)		26.1 (3.71)		17.4 (0.78)		16.9 (1.95)		93.9 (7.26)	
	>150 h	30.3 (1.52)		26.0 (1.00)		16.5 (2.00)		15.0 (3.60)		87.3 (1.52)	
Previous research training	None	31.3 (3.31)	0.441	26.5 (3.20)	0.376	16.8 (1.69)	0.927	16.4 (3.42)	0.987	90.9 (8.91)	0.842
	<40 h	31.0 (3.63)		28.2 (2.04)		16.8 (1.33)		16.7 (4.08)		92.7 (9.37)	
	≥40 h	32.8 (2.11)		25.8 (3.23)		17.0 (1.15)		16.4 (2.37)		92.1 (6.38)	
Reading or articles in the previous month	None	29.3 (4.16)	0.577	27.7 (3.21)	0.721	17.0 (1.73)	0.912	16.3 (0.57)	0.851	90.3 (9.01)	0.991
	1–3 articles	31.1 (3.38)		26.7 (3.22)		16.7 (1.68)		16.6 (3.18)		91.1 (8.62)	
	>3 articles	31.5 (3.19)		26.4 (3.16)		16.8 (1.64)		16.3 (3.55)		91.0 (8.95)	
Consultation of Twitter/Facebook/IG	Yes	31.7 (3.15)	0.006	26.9 (2.97)	0.013	16.9 (1.60)	0.032 *	16.6 (3.31)	0.344	92.1 (8.08)	0.016
	No	30.1 (3.36)		25.4 (3.52)		16.3 (1.72)		16.0 (3.62)		87.8 (9.99)	
Consultation of social networks	Never	30.2 (3.69)	0.268	24.9 (3.64)	0.377	16.0 (1.60)	0.081	15.4 (4.45)	0.242	86.5 (12.06)	0.444
	Occasionally	31.2 (2.74)		26.7 (2.91)		16.8 (1.39)		16.1 (3.18)		90.9 (7.38)	
	Monthly	30.3 (4.31)		26.7 (3.48)		16.4 (1.90)		16.9 (2.46)		90.2 (8.10)	
	Weekly	32.0 (2.87)		26.9 (2.91)		17.2 (1.36)		16.3 (3.61)		92.4 (8.15)	
	Daily	31.7 (3.77)		25.9 (3.67)		16.5 (2.46)		18.2 (2.80)		92.3 (11.02)	

* *p* < 0.05.

**Table 6:** Multivariable model for the explanatory variables of CS.

	Model	R2	Non-Standardized Coefficients		Standardized Coefficients	t	*p*	95% Confidence Interval for B	
			B	Error Dev.	Beta			Lower Limit	Upper Limit
CS Informative Comm.	Attitude EBP	0.313	2.602	0.577	0.347	4.506	<0.001	1.461	3.743
	EBP Knowledge		2.065	0.522	0.304	3.957	<0.001	1.034	3.096
CS Empathy	Special Admission	0.313	3.101	0.813	0.298	3.812	<0.001	1.491	4.711
	EBP Knowledge		2.269	0.571	0.355	3.972	<0.001	1.139	3.400
	Female		1.627	0.562	0.214	2.896	0.004	0.515	2.739
	Attitude EBP		1.384	0.617	0.192	2.242	0.027	0.163	2.606
CS Respect	EBP Knowledge	0.209	1.000	0.283	0.292	3.538	<0.001	0.441	1.558
	Attitude EBP		0.907	0.313	0.239	2.900	0.004	0.289	1.525
CS Assertiveness	Attitude EBP	0.028	1.304	0.624	0.168	2.091	0.038	0.072	2.536
Total CS	Attitude EBP	0.272	6.454	1.595	0.320	4.046	<0.001	3.302	9.606
	EBP Knowledge		5.219	1.441	0.287	3.621	<0.001	2.371	8.067

## Data Availability

The data presented in this study are available on request from the corresponding author. The data are not publicly available due to privacy or ethical restrictions.
